# A Systems Biology Study in Tomato Fruit Reveals Correlations between the Ascorbate Pool and Genes Involved in Ribosome Biogenesis, Translation, and the Heat-Shock Response

**DOI:** 10.3389/fpls.2018.00137

**Published:** 2018-02-14

**Authors:** Rebecca G. Stevens, Pierre Baldet, Jean-Paul Bouchet, Mathilde Causse, Catherine Deborde, Claire Deschodt, Mireille Faurobert, Cécile Garchery, Virginie Garcia, Hélène Gautier, Barbara Gouble, Mickaël Maucourt, Annick Moing, David Page, Johann Petit, Jean-Luc Poëssel, Vincent Truffault, Christophe Rothan

**Affiliations:** ^1^Institut National de la Recherche Agronomique, UR1052, Génétique et Amélioration des Fruits et Légumes, Montfavet, France; ^2^Institut National de la Recherche Agronomique, Université de Bordeaux, UMR1332, Biologie du Fruit et Pathologie, Villenave d'Ornon, France; ^3^Plateforme Métabolome du Centre de Génomique Fonctionnelle Bordeaux, Centre Institut National de la Recherche Agronomique de Bordeaux, Villenave d'Ornon, France; ^4^Institut National de la Recherche Agronomique, UR1115, Plantes et Systèmes de culture Horticoles, Avignon, France; ^5^Institut National de la Recherche Agronomique, Université d'Avignon et des Pays du Vaucluse, UMR408 Sécurité et Qualité des Produits d'Origine Végétale, Avignon, France

**Keywords:** ascorbate, cellular signaling, heat-shock response, redox, ribosome biogenesis, translation, tomato (*Solanum lycopersicum*)

## Abstract

Changing the balance between ascorbate, monodehydroascorbate, and dehydroascorbate in plant cells by manipulating the activity of enzymes involved in ascorbate synthesis or recycling of oxidized and reduced forms leads to multiple phenotypes. A systems biology approach including network analysis of the transcriptome, proteome and metabolites of RNAi lines for ascorbate oxidase, monodehydroascorbate reductase and galactonolactone dehydrogenase has been carried out in orange fruit pericarp of tomato (*Solanum lycopersicum*). The transcriptome of the RNAi ascorbate oxidase lines is inversed compared to the monodehydroascorbate reductase and galactonolactone dehydrogenase lines. Differentially expressed genes are involved in ribosome biogenesis and translation. This transcriptome inversion is also seen in response to different stresses in Arabidopsis. The transcriptome response is not well correlated with the proteome which, with the metabolites, are correlated to the activity of the ascorbate redox enzymes—ascorbate oxidase and monodehydroascorbate reductase. Differentially accumulated proteins include metacaspase, protein disulphide isomerase, chaperone DnaK and carbonic anhydrase and the metabolites chlorogenic acid, dehydroascorbate and alanine. The hub genes identified from the network analysis are involved in signaling, the heat-shock response and ribosome biogenesis. The results from this study therefore reveal one or several putative signals from the ascorbate pool which modify the transcriptional response and elements downstream.

## Introduction

Tomato has emerged as a model fruit for studying the relationships between ascorbate and fruit physiology. In addition to its nutritional value, ascorbate is a major antioxidant that contributes to fruit tolerance to biotic and abiotic stresses (Malacrida et al., 2006; Davey et al., 2007; Stevens et al., 2008). Studying the interactions between the ascorbate pool and fruit ripening is of particular interest because the vast majority of fresh market tomatoes produced in Europe are harvested at orange or light-red stages, before being fully ripe. At these stages, fruit undergo major modifications in color, firmness, sweetness, acidity, and aroma. These changes are under developmental and hormonal control and imply profound alterations in the transcriptome, proteome and metabolome (Klee and Giovannoni, 2011). In a previous study, we showed that chilling stress conditions (< 10°C) that can prevail during postharvest transport and storage affect fruit firmness, and that this effect is correlated with the ascorbate oxidation status (Stevens et al., 2008). However, the mechanisms by which ascorbate impinges on fruit physiology remain to be deciphered.

Ripening fruit are non-photosynthetic sink tissues relying on import of sucrose for providing reducing power, ATP, and precursor molecules necessary for synthesis of fruit-specific metabolites or proteins. The conversion of sucrose into hexose-phosphates is the starting point for glycolysis, the TCA cycle and the oxidative pentose phosphate pathway which provide the tissue with its metabolic and energetic requirements. The fruit therefore represents a complex biological system built on multi-layered interactions between metabolites, proteins and genes which form a network characteristic of the tissue. Induced genetic modifications are a good way of perturbing the system by varying the level of a gene and its corresponding protein and/or metabolites (network nodes) to evaluate the new steady state of the network, thus pinpointing the interactions that a particular node will have (Bassel et al., 2012; Krouk et al., 2013). A highly connected node (a hub) will share a number of edges with other nodes and perturbation of a regulatory hub will often give rise to highly pleiotropic phenotypes. This sort of approach is particularly useful to study a biological question as it can generate new hypotheses and identify regulatory genes.

Ascorbate is a major metabolite in plants including fruits with many functions often associated with its role in electron donation and as a cofactor (Gest et al., 2013b). Interestingly, although many studies in plants focus on the photo-protective or antioxidant functions of ascorbate, studies in animals have revealed further roles for ascorbate, for example the molecule functions as a cofactor for the ten-eleven translocation enzymes, which can convert 5-methylcytosine, to 5-hydroxymethylcytosine (5hmC) during demethylation of DNA in mouse embryonic stem cells (Blaschke et al., 2013). These enzymes are dioxygenases and therefore this is an example of a role for ascorbate as an electron donor to iron-dependent oxidoreductases. Ascorbate is also involved in oxidative protein folding in the endoplasmic reticulum and therefore plays a role in protein maturation (Banhegyi et al., 2003; Zito et al., 2012; Szarka and Lorincz, 2014). Dehydroascorbate has been shown to have a role in neuronal energy metabolism via activation of glucose-6-phosphate dehydrogenase thus increasing flux through the pentose phosphate pathway (Puskas et al., 2000; Cisternas et al., 2014). Furthermore, ascorbate has been identified as a kinase inhibitor (Carcamo et al., 2004) as well as an inhibitor of hexokinase (Fiorani et al., 2000) and its oxidized form can inhibit glyceraldehyde 3-phosphate dehydrogenase leading to an energetic crisis and cell death in cancer cells which uptake more dehydroascorbate via glucose transporters (Yun et al., 2015). These examples have not been shown in plants: the study of ascorbate in non-photosynthetic tissues may be key to revealing additional functions for the molecule; fruit is an ideal tissue, particularly in advanced ripening stages when photosynthetic activity is low.

Ascorbate's functions are profoundly affected by the oxidation state of the ascorbate pool: ascorbate is oxidized to the monodehydroascorbate radical, a relatively stable radical, which can regain an electron to regenerate ascorbate, or lose a second electron to become dehydroascorbate; these three forms are in equilibrium (Bors and Buettner, 1997). Plants also possess ascorbate oxidase enzymes which have roles in oxygen removal and signaling (De Tullio et al., 2007; De Tullio, 2012). Ascorbate oxidase activity has also been shown to have links with sugar metabolism and yield (Garchery et al., 2013), in a similar way to an isoform of monodehydroascorbate reductase (MDHAR), except that the phenotypes are inverted (Truffault et al., 2016): plants with reduced MDHAR activity show reduced growth and yield and lower levels of sugars (sucrose and hexose) particularly in leaves but counter-intuitively contain more ascorbate, particularly under light (Haroldsen et al., 2011; Gest et al., 2013a), whereas plants with reduced ascorbate oxidase (AO) activity have improved yield stability and more sucrose and hexose, particularly in leaves. A mitochondrial enzyme controls the final stage of ascorbate synthesis, galactonolactone dehydrogenase (GLD) (Alhagdow et al., 2007; Leferink et al., 2009), this enzyme is an integral part of complex I and is required for complex I function independently of ascorbate levels (Schimmeyer et al., 2016). Similarly to AO and MDHAR, manipulation of GLD levels in tomato RNAi lines leads to phenotypes including differences in ascorbate redox state, sugar metabolism, plant growth and fruit yield (Alhagdow et al., 2007).

**Figure 1:**
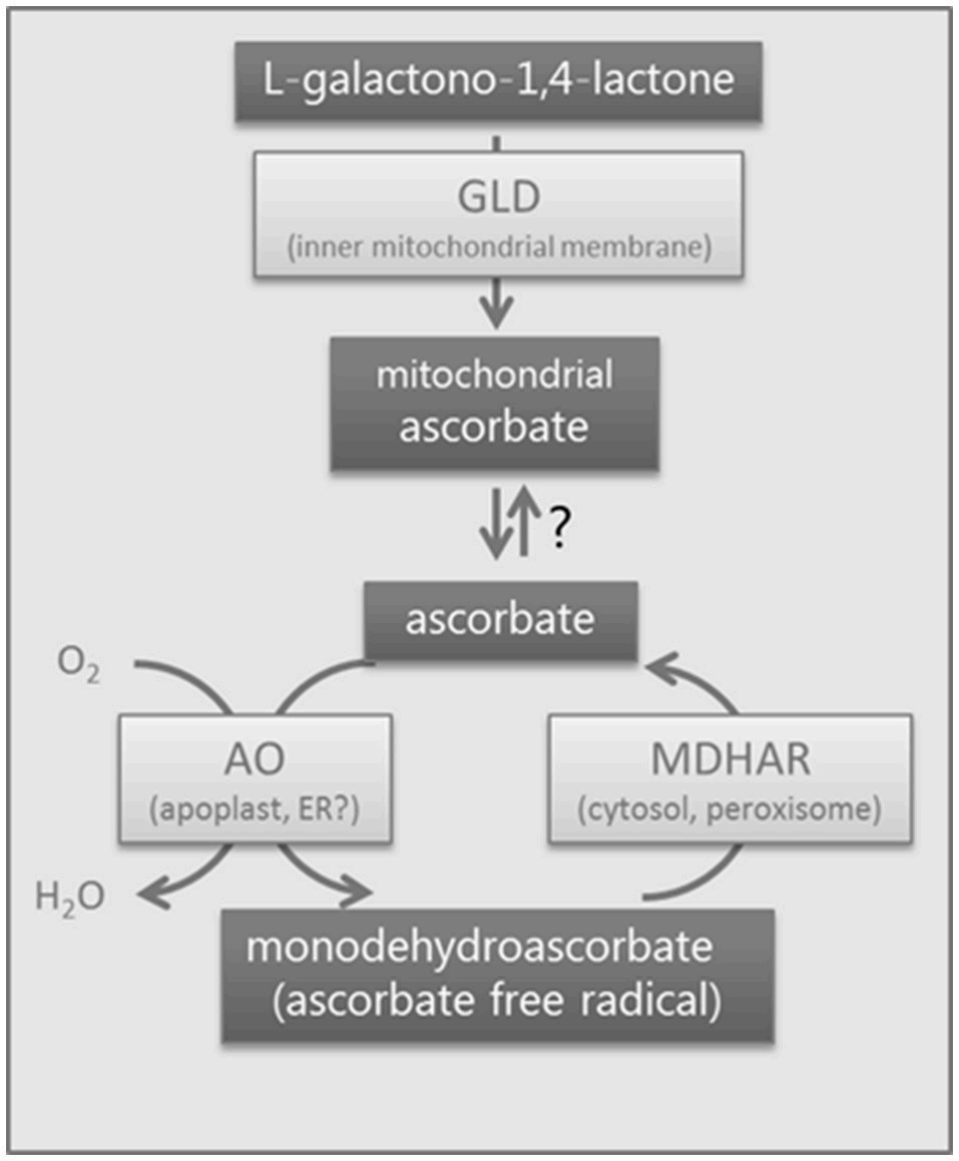
Biological system showing the enzymes studied. Schematic diagram of the enzymes studied in tomato using RNAi. GLD, galactonolactone dehydrogenase, a mitochondrial enzyme; AO, ascorbate oxidase, probably apoplastic; MDHAR, monodehydroascorbate reductase, a cytosolic and peroxisomal isoform. Ascorbate is generated in the mitochondrion and is transported into the cell via an unknown mechanism. Ascorbate oxidase oxidizes ascorbate to monodehydroascorbate which will disproportionate into dehydroascorbate and ascorbate. Monodehydroascorbate is recycled to ascorbate by enzymes with monodehydroascorbate reductase activity.

Modification of the ascorbate pool via manipulation of the activity of these enzymes has therefore generated many pleiotropic phenotypes at the physiological level. We hypothesize that the enzymes are affecting the levels of one or several metabolites, closely linked to their activity, which can be seen as a hub, involved in many key cellular processes. We have therefore carried out a large-scale study including phenotypic, metabolic, transcriptomic and proteomic data for orange fruit of RNAi lines for the three enzymes (AO, GLD, and MDHAR) shown in Figure 1 in order to identify their links with cellular metabolism and to correlate changes in gene expression, proteins and metabolites with the activity of these enzymes. A link is provided between the redox state of the ascorbate pool, cellular protein synthesis and stability, and ribosomal function.

## Materials and methods

### Greenhouse plant culture

West Virginia 106 cherry tomato plants were grown in a multi-span Venlo-type greenhouse in 5l pots (potting compost P3 Tref, Tref EGO substrates BV). Water and nutrients were supplied to the plants using a drip irrigation system to maintain 20–30% drainage. Light intensities of 300–700 photosynthetically active radiation were obtained over the culture period. Flowers were mechanically pollinated three times a week and side shoots removed as they appeared. The 35S-RNAi lines used have been previously described [AO, Solyc04g054690 (Garchery et al., 2013), GLD, Solyc10g079470 (Alhagdow et al., 2007), MDHAR (or MD), Solyc09g009390 (Gest et al., 2013a)]. In each case the specific transcript levels and enzyme activity were checked and shown to decrease. The individual lines chosen were the following: GLD5.3, AO15.1, and MD5 with a wild-type (WT) untransformed control. Plants homozygous for the transgene from the T2 generation were used. A total of 15 plants per line were used, 10 for fruit sampling and 5 for destructive phenotypic measurements. All 15 plants were used for non-destructive phenotypic measurements.

### Pre- and post-harvest phenotypic measurements

Plant height from the pot to the top of the apical meristem was measured weekly for 7 weeks on 10 plants per line. The growth rate was calculated as cm gained per day. The average leaf area of a fully expanded leaf (one leaf per plant, 10 plants) was calculated by scanning the leaf and calculating the area of the image using ImageJ software. Fruit characteristics (number of days between anthesis and orange maturity stage, and fruit weight) were calculated on at least six fruits per plant from 10 plants. Fruit weight, diameter, firmness measurements (compression and maximal force for skin rupture) and color were measured on a minimum of 20 fruits per line immediately after harvesting (ripe fruits). The weight loss of seven fruits per line, related to conservation properties, was measured after 5 days post-harvest at 20°C. Texture measurements were performed using a multi-purpose texturometer (TaPlus, Lloyd). The global firmness of each fruit was measured by a uniaxial compression test with a 50 mm flat disk (test-speed 10 cm.min^−1^) and expressed as the pressure (kPa) required for a 3% deformation of fruit height. The maximal force (N) corresponding to skin rupture was recorded using a flat cylinder tip (2 mm diameter) for a 7 mm penetration into the equatorial part of the fruit (test-speed 20 mm.min^−1^). The color was determined using a CR-400 chromameter (Minolta Co. Ltd., Osaka, Japan) and expressed in the CIE 1976 L^*^a^*^b^*^ Color Space coordinates (illuminant D65, 0_ view angle, illumination area diameter 8 mm). The chroma a^*^ axis is a good indicator for tomato ripening, varying from green (−60) to red (+60).

Statistical analysis on phenotypic characteristics was carried out using XLstat (Addinsoft, Paris, France) using a non-parametric comparison of means (Kruskal Wallis) with correction (Dunn). Different letters in the tables indicate significant differences (5% significance level).

### Experimental design for transcriptome and metabolome analyses

Fruit were tagged at anthesis and harvested 20 days later (for ascorbate measurements and proteome analysis only) or when orange. Fruit were harvested during the growing season (from September to November) from trusses 2, 3, and 4 from 10 plants using three fruit/truss/plant to make 3 pools of 30 fruits per line. Fruit pericarp including the epidermis was retained, frozen in liquid nitrogen and stored at −80°C before being ground to a fine powder in liquid nitrogen for the following analyses.

### NMR metabolomic profiling

Polar metabolites were extracted from lyophilized powder with a series of hot ethanol/water solutions and quantified by ^1^H-NMR as previously described (Mounet et al., 2007). For extract preparation and NMR acquisition parameters, special care was taken to allow absolute quantification of individual metabolites through addition of 5 mM ethylene diamine tetra-acetic acid disodium salt solution to improve the NMR signal resolution and quantification of organic acids, adequate choice of the NMR acquisition parameters, and use through an electronic reference for quantification (Moing et al., 2004). Unknown compounds were quantified in arbitrary units. The lyophilized extracts were titrated with KOD to apparent pH 6 in 400 mM potassium phosphate buffer solution in D_2_O and lyophilized again. The ^1^H-NMR spectra were recorded on each dried titrated extract solubilized in 0.5 mL D_2_O with the sodium salt of (trimethylsilyl)propionic-2,2,3,3-d4 acid (TSP) at a final concentration of 0.01% for chemical shift calibration, at 500.162 MHz on an Avance spectrometer (Bruker BioSpin, Wissembourg, France) using a 5 mm inverse probe. Sixty-four scans of 32K data points were acquired with a spectral width of 6000 Hz, acquisition time of 2.73 s, 90° pulse angle and recycle delays of 25 s. The raw ^1^H NMR spectral profiles have been deposited, with associated metadata, into the Metabolomics Repository of Bordeaux MeRy-B (http://services.cbib.u-bordeaux2.fr/MERYB/public/PublicREF.php?REF=T07001).

### Individual metabolites

Ascorbate and dehydroascorbate concentrations in fruit powder were analyzed according to previous protocols based on the reduction of an iron-dipyridyl complex leading to a color change detectable spectrophotometrically with a filter at 525 nm (Stevens et al., 2006) with modifications (Garchery et al., 2013).

Individual polyphenols were analyzed using 200 mg of lyophilised powder from the orange fruit (3 pools), to which of 25 μL of taxifolin (1 mg.mL^−1^, Extrasynthèse, Genay France) was added as an internal standard. Three successive extractions with 70% ethanol were carried out before evaporation to dryness in a vacuum concentrator. The pellet was resuspended in 300 μL water, 700 μL methanol and filtered with a PTFE syringe filter. Ten micro liters were analyzed by reversed-phase HPLC (Shimadzu Prominence system coupled with a Diode Array Detector (HPLC-DAD), Merck LichroCART® 250-4 column; phase Superspher® 100 RP-18 end capped 4 μm) using a gradient elution with ortho-phosphoric acidified water (pH 2.6) (A) and methanol (B) as solvents. The program was as follows: 0 min 15% B, 10 min 15% B, 20 min 21% B, 32 min 21% B, 42 min 23% B, 52 min 23% B, 107 min 60% B; flow rate 0.5 mL.min^−1^ from 0 to 44 min, 0.5 mL.min^−1^ to 0.7 mL.min^−1^ from 44 to 46 min, 0.7 mL.min^−1^ from 46 to 60 min. Phenolic compounds were monitored at 280 nm. Chlorogenic acid and rutin were quantified using external calibration curves of standard compounds (Extrasynthèse, Genay, France); other compounds were expressed in arbitrary units.

### Proteome analysis

Protein extraction, two-dimensional gel electrophoresis, and protein identification and classification were carried out as previously described (Xu et al., 2013). Briefly, proteins were extracted using the phenol extraction method developed by Faurobert et al. (2007). Proteins were then separated by two-dimensional gel electrophoresis. After Coomassie colloidal staining, image analysis was performed with Samespot software (version 4.1), and the normalized spot volumes were obtained. Of the 108 spots revealed, 49 were eliminated as not being variable. Protein identification of the 59 variable spots was performed at the proteome platform of Le Moulon (Gif-sur-Yvette) using a nano-LC-MS/MS method following a previously described procedure (Xu et al., 2013). The database search was run against the tomato unigene database version 4 held by the Solanaceae Genome Network (http://solgenomics.net/; Tomato_200607_build_2) with X!Tandem software (http://www.thegpm.org/TANDEM/, version 2010.12.01.1). Sequences were subsequently converted to Solycs (ITAG version 2.4) by Blast analysis. This conversion was confirmed by re-analyzing the mass spectra or by searching for similarity between the protein peptide sequences and the ITAG2.4 version of the genome by TBLASTN.

### Microarray analysis and comparison of transcriptome data

Microarray experiments were performed on the three biological replicates (3 pools) and technical duplicates with the dyes reversed (dye swap). The TOM2 cDNA microarray [CGE, Boyce Thompson Institute, Cornell University, http://bti.cornell.edu/CGEP/CGEP.html, (Fei et al., 2011)] containing 12,160 70-mer oligonucleotides corresponding to 11,862 unique and randomly selected transcripts of the tomato genome were used. Sample preparation and microarray hybridization and analysis were carried out as described (Mounet et al., 2009). Briefly, one μg of DNA-free total RNA was amplified with the MessageAmpTM aRNA Ambion Kit (Ambion/Applied Biosystems), labeled using the CyScribe Post-Labeling Kit (GE Healthcare) and the cy3- and cy5-labeled cDNAs were hybridized to TOM2 microarrays using an automatic hybridization station HS 4800 (Tecan) as described. Microarray slides were scanned with a Genepix 4000 B fluorescence reader (Axon Instruments) using Genepix 4.0 Pro image acquisition software. The photomultiplier tube voltage was adjusted to 640 V for Cy3 and 700 V for Cy5. Spot flagging was carried out by Genepix (missing spots) and by visual inspection of the images in order to exclude saturated and heterogeneous spots. The raw data were then submitted to LIMMA R Package v2.12.0 (Ritchie et al., 2015) for data visualization, normalization as described (Alhagdow et al., 2007). Background was first subtracted from the raw median intensity values using the normexp function. Two normalization algorithms were successively applied to the raw data (Yang et al., 2002): print-tip loess (default parameters) as the within-array normalization, followed by a quantile normalization (default parameters) as between-array normalization. The normalized data were then analyzed as described below. To convert to current Solyc identifiers, each of the TOM2 probes was blasted against the tomato genome (ITAG2.3, minimum alignment length 40 bp, minimum identity 84%), which had been sequenced in the meantime, and following this 7633 unique Solycs were retained.

### R/maanova

Differential expression and transcriptome comparisons were carried out using the R/maanova package [https://www.bioconductor.org/packages/release/bioc/html/maanova.html (R Development-Core-Team, 2015)]. Means and standard errors were calculated for each gene in each line. A simple model taking into account the genotype (line) was used. A matrix of comparisons was used for the pairwise comparison of the transcriptome datasets first of each transgenic line with WT and then with each other. For each comparison, a threshold for the FDR corrected *p*-value of 0.001 was applied. Following initial tests, we added a sub-hypothesis to the comparison matrix to test whether the AO-GLD transcriptome and the AO-MDHAR transcriptomes were inverted i.e., the wild-type expression was the average of the gene expression in the two transgenic lines being compared. Expression differences were expressed as log2 of the Fold Change between the two lines. The linear model was tested using 1,000 iterations and then an FDR adjustment of the *p*-values obtained. A threshold for the corrected *p*-value of 0.001 was set to identify the core gene set for the network visualization.

### Weighted-gene-correlation network analysis

Weighted gene correlation network analysis (WGCNA) was carried out using the normalized expression data for the 7633 genes with a “trait” file including the 22 metabolites and 40 proteins using the WGCNA package in “R” (Langfelder and Horvath, 2008). No outliers were detected after sample clustering so the whole dataset was used. A soft threshold of 14 and minimum module size of 25 were set. Networks were visualized using Cytoscape version 3.2.0 (Cline et al., 2007) (www.cytoscape.org) using the node and edge output file from the WGCNA package with a threshold set to 0.52 (to fix a number of nodes and edges suitable for visualization). The dataset used contained the 182 differentially expressed genes (differentially expressed in the pairwise comparisons of the three types of transgenic lines), the 22 metabolites and 40 proteins. The number of genes was limited to facilitate network creation and to give weight to the proteins and metabolites. The layout was first designated as degree sorted circle layout and then a degree layout was chosen to aid node identification. Node size is proportional to the degree of connectivity.

### Gene ontology

Gene ontology (GO) enrichment on the whole transcriptome signatures generated from pairwise comparisons of the lines was carried out using the R package “goseq” (Young et al., 2010) with the corrected *p*-value false detection rate set to 5%. The package requires the log of fold-change and *p*-value from the R/maanova analysis. No length correction was required as the data was from microarrays. Tomato gene ontology for the 182 differentially expressed genes was carried out using the online tools available from the Boyce Thompson Institute, Cornell University (http://bioinfo.bti.cornell.edu/cgi-bin/MetGenMAP/home.cgi, Boyle et al., 2004).

### ROSMETER

The ROSMETER bioinformatic tool allows evaluation of transcriptome signatures linked to reactive oxygen species (ROS) (Rosenwasser et al., 2013). The tool consists of a web-based interface whereby vector-based correlations between stored transcriptome data, chosen for its ROS signature, and the provided data of interest are calculated. The stored data are microarray-based data from wild type and Arabidopsis mutants for the following genes: ascorbate peroxidase, the *flu* mutant, mitochondrial alternative oxidase, catalase 2 and superoxide dismutase subjected or not to the following stresses: ozone, high light, hydrogen peroxide, drought, rotenone, methyl viologen and 3-aminotriazole as described in the original research paper. The data from the RNAi lines normalized to wild-type (fold change and *p*-value) were compared with this series of transcriptomes arising from different conditions or mutations in *Arabidopsis thaliana*. In order to compare the transcriptomes, the tomato Solycs were first converted to their closest Arabidopsis homolog and submitted to the ROSMETER interface. In the heatmaps, red represents a positive correlation and green a negative one.

## Results

### Transgenic lines show slight differences in growth and development of plants and fruit

The three RNAi transgenic lines (AO: ascorbate oxidase; GLD: galactonolactone dehydrogenase and MDHAR: monodehydroascorbate reductase) and wild-type plants grown in the greenhouse were subjected to simple phenotypic analyses to measure plant height, growth rate and final leaf area, the duration of fruit ripening (anthesis to orange fruit) and fruit weight were also measured. The results for the plant phenotypes are shown in Table 1: AO and GLD RNAi lines had a slower growth rate and a smaller final plant height compared to wild-type plants. Fruit from the same two transgenic lines also took longer to reach maturity (Table 2). Fruit from the MDHAR silenced lines were significantly smaller than fruit from the other lines, including wild-type (Tables 2, 3), as per previous results (Truffault et al., 2016). Fruits from the GLD RNAi line were also smaller in diameter (Table 3). Harvested fruits were analyzed for fruit firmness, color, and post-harvest weight loss (Table 3). No significant differences were found between the lines in terms of compression or color but the AO and MDHAR lines required less force to penetrate the skin compared to wild type. MDHAR RNAi fruit also showed greater post-harvest weight-loss compared to the WT, AO, and GLD lines.

**Table 1:** Plant phenotypes of transgenic lines and WT.

	**Height after 7 days (cm)**	**SE**	**p**	**Height after 7 weeks (cm)**	**SE**	**p**	**Starting growth rate (cm/day)**	**SE**	**p**	**Final growth rate (cm/day)**	**SE**	**p**	**Average leaf area (cm^2^)**	**SE**	**p**
WT	26.2	3.7	a	193.0	9.0	b	2.7	0.2	b	5.84	0.32	b	313	46	a
AO	23.7	1.8	a	181.8	4.9	ab	2.5	0.2	ab	5.74	0.27	ab	296	49	a
GLD	24.5	2.3	a	171.3	6.3	a	2.4	0.1	a	5.22	0.31	a	348	10	a
MDHAR	25.3	2.2	a	191.8	14.0	b	2.7	0.3	b	6.06	0.79	b	326	37	a

*Measurements were carried out on greenhouse-grown plants as described in the Materials and methods. Data show means from 10 plants per line with standard error (SE). A comparison of means was carried out using a Kruskal Wallis test with correction (Dunn). Different letters indicate significant differences (5% significance level)*.

**Table 2:** Fruit phenotypes of transgenic lines and WT.

	**Days to orange**	**SE**	**p**	**Individual fruit weight (g)**	**SE**	**p**	**% Dry weight (orange)**	**SE**	**p**
WT	40.3	2.1	a	6.3	1.2	a	7.1	0.1	a
AO	41.9	1.5	b	6.1	0.8	a	7.0	0.1	a
GLD	42.7	1.4	b	6.0	1.0	a	6.8	0.1	a
MDHAR	40.7	1.8	a	3.8	0.9	b	7.0	0.1	a

*Measurements were carried out on greenhouse-grown plants as described in the Materials and methods. Data show means from at least 6 fruits per plant of 10 plants per line with standard error (SE). A comparison of means was carried out using a Kruskal Wallis test with correction (Dunn). Different letters indicate significant differences (5% significance level)*.

**Table 3:** Fruit physical characteristics of transgenic lines and WT at harvest.

	**Fruit weight (g)**	**SE**	**p**	**Diameter (mm)**	**SE**	**p**	**Compression (kPa)**	**SE**	**p**	**Max force—skin rupture (N)**	**SE**	**p**	Color (a^*^)	**SE**	**p**	**Postharvest weight loss (%)**	**SE**	**p**
WT	9.3	0.4	a	25.7	0.4	a	49.5	2.5	a	5.7	0.2	a	10.3	1.0	a	0.043	0.001	a
AO	8.3	0.4	a	24.4	0.5	ab	43.1	3.0	a	4.6	0.2	b	13.8	1.6	a	0.049	0.003	ab
GLD	7.8	0.4	a	23.6	0.5	b	49.4	4.0	a	5.4	0.3	ab	13.1	1.7	a	0.046	0.001	a
MDHAR	5.3	0.3	b	21.1	0.4	c	49.9	2.6	a	4.6	0.3	b	15.7	1.7	a	0.063	0.002	bc

Fruit physical characteristics were measured on red-ripe fruit of at least 20 fruit per line as described in the Materials and methods. Measurements show means with standard error (SE). A comparison of means was carried out using a Kruskal Wallis test with correction (Dunn). Different letters indicate significant differences (5% significance level). a^*^ is the name of the color scale.

### Metabolic differences are seen in phenolic and amino acid metabolism

**Figure 2:**
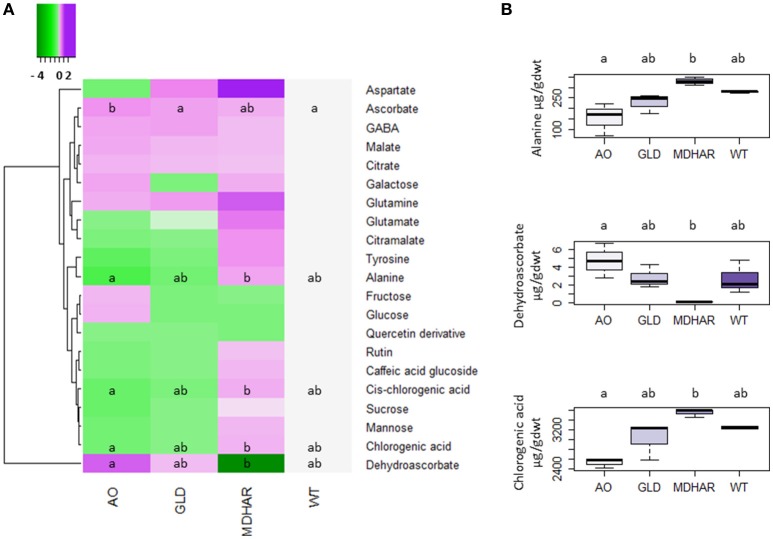
Accumulation of metabolites in orange pericarp fruit (3 biological replicates of 30 fruits) of the transgenic lines and wild-type. All metabolites except for ascorbate, dehydroascorbate and the polyphenols which were analyzed by specific methods, were analyzed by ^1^H-NMR. **(A)** Heatmap showing a log2 of the metabolite ratio with the wild-type for each transgenic line. The scale goes from green (metabolite decreased compared to wild-type) to purple (metabolite increased). A comparison of the means was carried out using a Kruskal Wallis test with correction (Dunn). Different letters indicate significant differences (5% significance level). **(B)** Boxplots showing raw data for three differentially accumulated metabolites in the transgenic lines and wild-type: alanine, dehydroascorbate and chlorogenic acid. A comparison of the means was carried out using a Kruskal Wallis test with correction (Dunn). Different letters indicate significant differences (5% significance level).

Metabolome analysis was carried out on the fruit pericarp samples using ^1^H-NMR profiling of polar extracts. Ascorbate (Table 4) and major fruit polyphenols (Table 5) were analyzed by specific methods. Ascorbate was significantly increased in orange fruit pericarp of MDHAR lines as previously observed (Gest et al., 2013a) and dehydroascorbate was significantly different in AO and MDHAR lines, although this difference was the opposite of what would be expected given the activities of the enzymes; GLD and WT had intermediate values. Ascorbate data for fruit 20 days after anthesis is presented in Supplementary Table S1, unlike orange fruit, no significant differences are seen. The polyphenol that varied most in the transgenic lines was chlorogenic acid, which is also the most prevalent polyphenol, and its stereoisomer cis-chlorogenic acid: as for dehydroascorbate, levels were significantly different in AO and MDHAR lines, AO lines containing less chlorogenic acid than wild-type whereas the MDHAR lines contained more (Table 5 and Figure 2). Levels of these polyphenols in the GLD RNAi fruit were not significantly different to wild-type. The metabolites detected and quantified by ^1^H-NMR are shown in Figure 2 (and Supplementary Table S2). Metabolites that differentiate the lines included the amino acids alanine and tyrosine which were highest in MDHAR and lowest in AO lines (significant for alanine) and the hexose sugars glucose and fructose which showed a tendency in contrast to the aforementioned amino acids to be highest in AO lines.

**Table 4:** Ascorbate and dehydroascorbate levels in orange fruit pericarp of transgenic lines and WT.

	**Reduced AsA (mg/100gfwt)**	**SE**	**p**	**DHA (mg/100gfwt)**	**SE**	**p**
WT	15.46	1.30	a	2.71	1.39	ab
AO	18.99	1.97	ab	4.73	1.32	a
GLD	19.19	0.65	ab	2.83	0.97	ab
MDHAR	21.72	1.05	b	0	0.68	b

*Ascorbate and dehydroascorbate were assayed in the pericarp tissue (3 biological replicates of 30 fruits per pool) of the lines by a spectrophotometric method. Measurements show means with standard error (SE). A comparison of means was carried out using a Kruskal Wallis test with correction (Dunn). Different letters indicate significant differences (5% significance level)*.

**Table 5:** Polyphenols in orange fruit pericarp of transgenic lines and WT.

	**Chlorogenic acid (μg/gdwt)**	**SE**	**p**	**Caffeic acid glucoside (arbitrary units)**	**SE**	**p**	**Cis-chlorogenic acid (arbitrary units)**	**SE**	**p**	**Quercetin derivative (arbitrary units)**	**SE**	**p**	**Rutin (μg/gdwt)**	**SE**	**p**
WT	3245.2	31.2	ab	3.5	0.0	a	1.3	0.0	ab	0.4	0.00	a	435.5	2.9	a
AO	2537.5	81.5	a	3.0	0.2	a	1.0	0.1	a	0.4	0.02	a	397.1	8.1	a
GLD	3029.0	298.5	ab	3.2	0.5	a	1.1	0.1	ab	0.4	0.01	a	406.0	21.4	a
MDHAR	3554.4	68.3	b	3.7	0.2	a	1.5	0.1	b	0.3	0.01	a	446.7	21.3	a

*Polyphenolic compounds were assayed in the pericarp tissue (3 biological replicates of 30 fruits per pool) of the lines by HPLC-DAD. When the purified compound was not available for exact quantification, arbitrary units corresponding to peak surface were used. Measurements show means with standard error (SE). A comparison of means was carried out using a Kruskal Wallis test with correction (Dunn). Different letters indicate significant differences (5% significance level)*.

### The fruit proteome is inversely affected by MDHAR and AO expression

**Figure 3:**
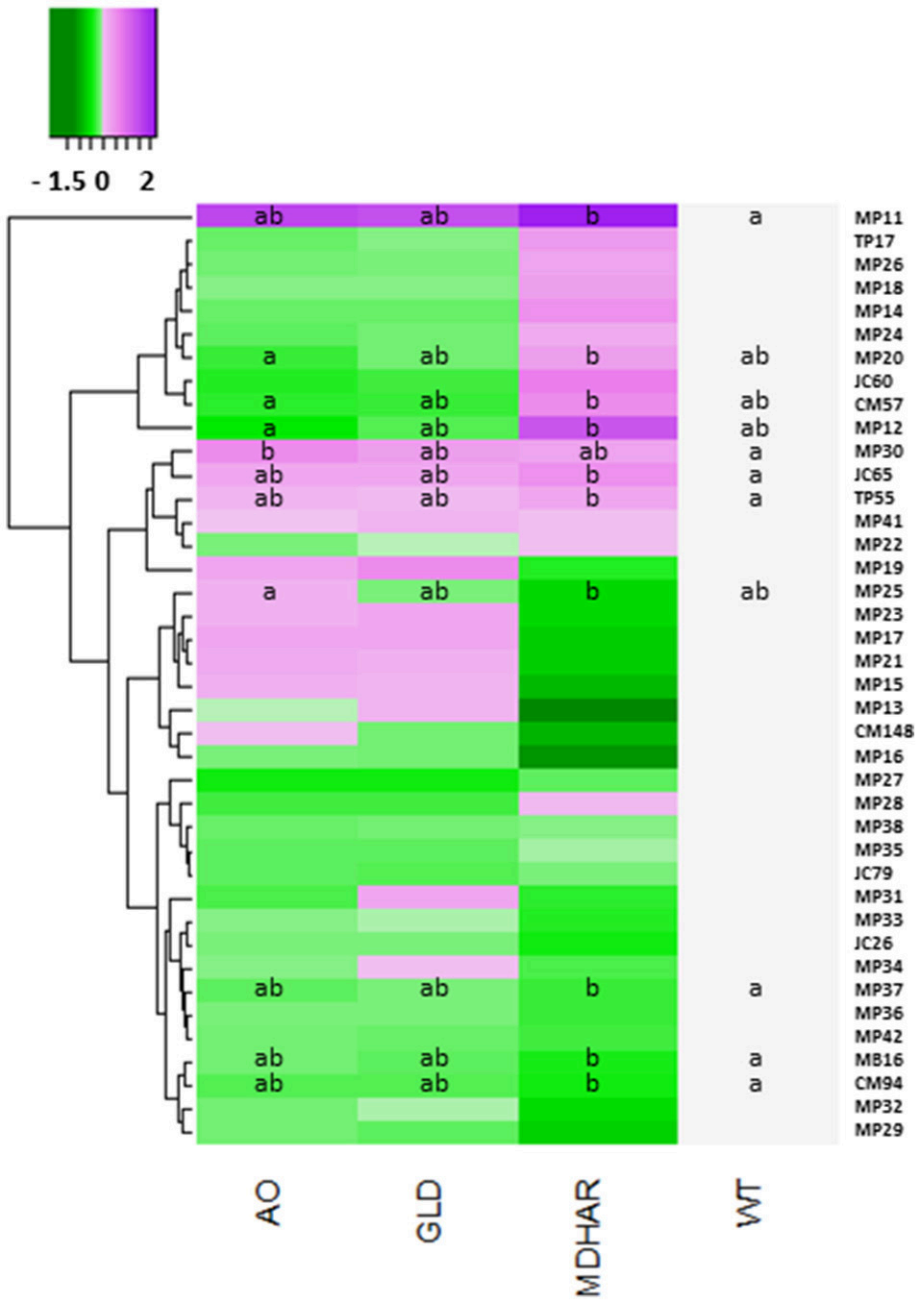
Heatmap showing protein levels in orange pericarp fruit (3 biological replicates of 30 fruits) of the transgenic lines and wild-type. All proteins were separated by two-dimensional gel electrophoresis and identified by mass spectrometry. The log2 of the protein ratio with the wild-type for each transgenic line is presented. The scale goes from green (protein decreased compared to wild-type) to purple (protein increased). A comparison of the means was carried out using a Kruskal Wallis test with correction (Dunn). Different letters indicate significant differences (5% significance level). The proteins are identified by their Spot reference ID, the correspondence for which can be found in Table 6.

The differentially expressed protein spots led to the identification of 34 proteins by mass spectrometry (Table 6). Differences between the lines are presented as a heatmap of the log2 of the ratio with the WT (Figure 3; Table 6, raw data in Supplementary Table S3). The line most affected in terms of its fruit proteome is MDHAR, for which up-regulated proteins included a 60S ribosomal protein, a class I heat-shock protein and a cysteine protease inhibitor. Down-regulated proteins included a heat-shock protein, (hsp70 homolog), lactoylglutathione lyase and an amidase hydantoinase/carbamoylase family protein. The proteome of the GLD line did not show significant differences to the wild-type, despite visually resembling the ascorbate oxidase profile. A number of proteins showed opposite regulation in AO and MDHAR lines, these included chaperone DnaK (MDHAR down-regulated, AO up-regulated), a probable carbonic anhydrase, a protein disulphide isomerase and a metacaspase 7, a protein with cysteine endopeptidase activity and a positive mediator of cell death which is AO down-regulated and MDHAR up-regulated. One protein was up-regulated in the AO line which is an F1F0-ATPase inhibitor protein. The data for fruit 20 days after anthesis is shown in Supplementary Figure S1: only two proteins showed statistically significant differences in spot volumes between wild-type and the MDHAR line.

**Table 6:** Proteins in orange fruit pericarp of the lines as identified by mass spectrometry.

**Spot ID**	**ITAG2.4**	**Annotated function**	**Significant difference in lines**	**Fold change of transcript AO vs. MDHAR**	***p*-value (FDR)**	**Correlation transcript-protein**
CM148	Solyc01g099190.2.1	Lipoxygenase		No data	No data	
MP25	Solyc01g103450.2.1	Chaperone DnaK	MDHAR down, AO up			
MP26	Solyc01g104920.2.1	26S protease regulatory subunit 8 homolog		−1.84	0.002	
MP14	Solyc01g111300.2.1	Cold shock protein-1				
MP31	Solyc02g070510.2.1	Proteasome subunit alpha type		No data	No data	
MB16	Solyc02g080630.2.1	Lactoylglutathione lyase	MDHAR down			
MP18	Solyc02g085790.2.1	T-complex protein 1 subunit zeta		5.82	0.005	
MP38	Solyc02g091100.2.1	Oxalyl-CoA decarboxylase				
MP42	Solyc02g092670.1.1	Subtilisin-like protease		No data	No data	
JC65	Solyc03g097270.2.1	Cysteine proteinase inhibitor	MDHAR up	−1.40	0.017	Yes
MP24	Solyc04g045340.2.1	Phosphoglucomutase				
MP29	Solyc04g045340.2.1	Phosphoglucomutase				
CM57	Solyc05g005490.2.1	Carbonic anhydrase	MDHAR up, AO down	1.98	0.008	No
MP17	Solyc05g012580.1.1	Unknown Protein				
MP11	Solyc05g054580.2.1/Solyc09g091520.1.1	60S acidic ribosomal protein P0	MDHAR up	−1.34	0.023	Yes
JC26	Solyc06g005160.2.1	Ascorbate peroxidase		1.25	0.036	
MP13	Solyc06g005940.2.1	Protein disulfide isomerase				
MP33	Solyc06g005940.2.1	Protein disulfide isomerase				
JC60	Solyc06g059740.2.1	Alcohol dehydrogenase 2		1.49	0.004	
TP17	Solyc06g059740.2.1	Alcohol dehydrogenase 2		1.49	0.004	
MP20	Solyc06g060290.2.1	Protein disulfide isomerase	MDHAR up, AO down	No data	No data	
MP36	Solyc06g065270.2.1	Adenylate kinase				
TP55	Solyc06g076520.1.1	Class I heat shock protein	MDHAR up			
CM94	Solyc07g005820.2.1	Heat shock protein 70	MDHAR down			
MP23	Solyc07g066600.2.1	Phosphoglycerate kinase		−1.36	0.033	
MP22	Solyc08g014130.2.1	2-isopropylmalate synthase 1		2.05	0.000	
JC79	Solyc08g014130.2.1	2-isopropylmalate synthase 1		2.05	0.000	
MP21	Solyc08g062660.2.1	Ran GTPase binding protein		No data	No data	
MP41	Solyc09g082060.2.1	Cysteine synthase				
MP37	Solyc09g083410.2.1	Amidase hydantoinase/carbamoylase family protein	MDHAR down	No data	No data	
MP19	Solyc09g090330.2.1	Harpin binding protein 1		No data	No data	
MP12	Solyc09g098150.2.1	Metacaspase 7	MDHAR up, AO down	2.01	0.003	No
MP16	Solyc09g098150.2.1	Metacaspase 7		2.01	0.003	
MP32	Solyc09g098150.2.1	Metacaspase 7		2.01	0.003	
MP34	Solyc10g081240.1.1	Protein grpE				
MP28	Solyc10g086580.1.1	Ribulose-1 5-bisphosphate carboxylase/oxygenase activase 1				
MP27	Solyc11g006970.1.1	Unknown protein DS12 from 2D-PAGE of leaf, chloroplastic		No data	No data	
MP30	Solyc11g068510.1.1	F1F0-ATPase inhibitor protein	AO up	No data	No data	
MP35	Solyc11g069000.1.1	T-complex protein 1 subunit beta		−1.46	0.035	
MP15	Solyc12g056230.1.1	Glutathione peroxidase		No data	No data	

*The predicted Solyc (https://solgenomics.net/organism/Solanum_lycopersicum/genome; version 2.4 of the International Tomato Annotation Group, ITAG) is shown with the annotated function and significant difference between lines where applicable (5% significance level). The fold-change and p-value of the corresponding transcript, when present on the microarray and for p-values of < 0.05, is also shown for the AO-MDHAR lines and whether this fold-change is correlated with the difference seen in protein levels*.

### Weighted-gene correlation network analysis reveals clusters of genes associated with both metabolites and proteins

The normalized transcriptome data for the four lines was subjected to a weighted-gene correlation network analysis to identify clusters of correlated genes and compare them with different phenotypes (metabolite and protein levels). We identified 38 modules of between 26 and 851 genes (Supplementary Figure S2). The number of genes in each module was too small to give significant differences following GO enrichment analysis but revealed co-regulation of metabolites or proteins with genes in modules or groups of modules. For example, the amino acids were positively correlated with the gene modules “dark red,” “blue,” “green-yellow” and “yellow.” The full data set with module membership is found in Supplementary Table S4.

### Pairwise comparison of transcriptome data reveals the same set of differentially regulated genes in the comparison AO-MDHAR and AO-GLD

**Figure 4:**
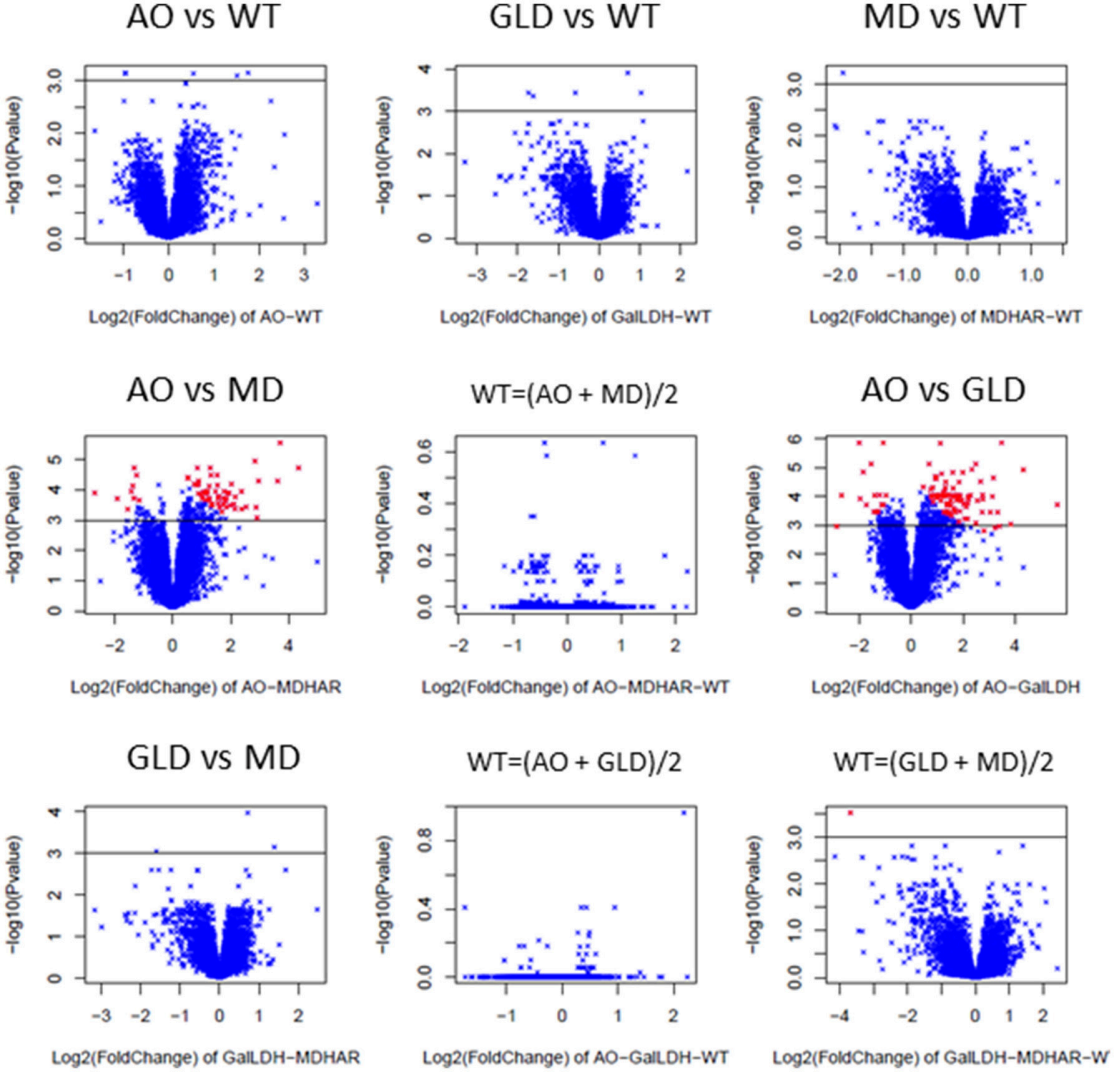
Pairwise transcriptome comparisons as illustrated using volcano plots (log2 of fold change plotted against –log10 of the *p*-value for the differential expression). The expression of the 7633 genes from the microarray analysis of RNA extracted from the orange fruit pericarp (3 biological replicates of 30 fruit) of the different lines was compared with technical duplicates (dyes swapped). The comparisons were carried out first between the transgenic lines (GalLDH = GLD) and wild-type (AO-WT, comparison 1; GalLDH-WT, comparison 2; MDHAR-WT, comparison 3) before comparing the transgenic lines between themselves (AO-MDHAR, comparison 4, AO-GalLDH, comparison 6, and GalLDH-MDHAR, comparison 7). Afterward the hypothesis was tested that the WT expression was the average of the expression in two given transgenic lines (see Materials and methods; WT = average AO and MD, comparison 5, WT = average AO and GalLDH, comparison 8, WT = average GalLDH and MDHAR, comparison 9). The black line is drawn to represent a threshold *p*-value of 0.001, the red points represent differentially expressed genes.

Each of the three transgenic lines was compared with wild-type and then the lines were compared two by two. For the pairwise comparisons of the transgenic lines, the hypothesis that the gene expression in the wild-type was the average of the gene expression in each transgenic line was tested using the maanova model (see Materials and methods). Volcano plots for each pairwise comparison showing the log2 of the fold-change in gene expression between the lines and the associated *p*-value (−log10 transformed) are shown in Figure 4. The AO-MDHAR and AO-GLD comparisons generated the highest number of differentially expressed genes (comparisons 4; 290 genes and comparison 6; 330 genes). The comparisons WT = (transgenic X + transgenic Y)/2 are comparisons 5, 8, and 9. If the hypothesis is true then the *p*-values become non-significant and the −log10 of the *p*-values gives low values which is the case for the comparisons 5 [WT = (AO + MDHAR)/2] and 8 [WT = (AO + GLD)/2] but not the case for the comparison 9 [WT = (GLD + MDHAR)/2]. The whole dataset including means, standard error, *p* and *F*-values and the fold-change with *p*-values for each comparison is available in Supplementary Table S5.

### The differentially expressed genes are enriched for functions involving translation and ribosome-biogenesis

The AO-MDHAR and AO-GLD comparisons generated the highest number of differentially expressed genes and the expression in the WT is always intermediate. Comparison of these two gene sets showed 182 genes common to each comparison, defining a core set of genes for further analysis. Independently of the comparison and the direction of change, differentially expressed genes were part of one “molecular function” category: structural constituent of ribosome (GO:00037) and two “biological process” categories: ribosome biogenesis and translation (GO:00422 and GO:00064). These genes were up-regulated in the AO line and down-regulated in the MDHAR and GLD RNAi lines. The core set of 182 genes were analyzed using the *Solanaceae* Genome Network GO-enrichment tool and this analysis gave further categories associated with translation, ribosomes, unfolded protein binding, GTPase regulator activity and nucleotide binding. The categories “structural constituent of ribosome,” “small GTPase regulator activity” and “oxidoreductase activity acting on NADH or NADPH” were more than ten-fold enriched in the cluster compared to the genome (Table 7).

**Table 7:** Gene ontology enrichment on the 182 set of common genes using the tools available at Boyce Thompson Institute, Cornell University (http://bioinfo.bti.cornell.edu/cgi-bin/MetGenMAP/home.cgi).

**Gene ontology term**	**Number of genes**	**Cluster frequency (%)**	**Genome frequency (%)**	**Corrected *p*-value**
Unfolded protein binding	3	1.6	0.2	0.038
Structural molecule activity	11	6.0	0.7	0.000
Structural constituent of ribosome	10	5.5	0.5	0.000
Small molecule binding	16	8.8	3.9	0.028
Small GTPase regulator activity	3	1.6	0.1	0.032
Ribonucleotide binding	11	6.0	2.0	0.025
Purine ribonucleotide binding	11	6.0	2.0	0.023
Purine ribonucleoside triphosphate binding	11	6.0	2.0	0.025
Purine nucleotide binding	11	6.0	2.0	0.021
Protein binding	44	24.2	11.9	0.000
Oxidoreductase activity acting on NADH or NADPH	4	2.2	0.2	0.003
Oxidoreductase activity	16	8.8	2.8	0.000
Organic cyclic compound binding	16	8.8	3.3	0.013
Nucleotide binding	16	8.8	3.3	0.015
Nucleoside phosphate binding	16	8.8	3.3	0.012
Catalytic activity	53	29.1	17.9	0.000
Binding	79	43.4	25.1	0.000
ATP binding	9	4.9	1.4	0.020
Adenyl ribonucleotide binding	9	4.9	1.4	0.019
Adenyl nucleotide binding	9	4.9	1.4	0.020

*The table shows the cluster frequency (the 182 genes) compared to the genome frequency for each category with the FDR-corrected p-value. The three categories (structural constituent of ribosomes, small GTPase regulator activity and oxidoreductase activity acting on NADH or NADPH) are where there is the biggest difference between the genome frequency and the cluster frequency*.

### The subset of 182 genes defines a regulatory network with several hub genes including heat-shock transcription factors, chaperones, and ribosomal proteins

**Figure 5:**
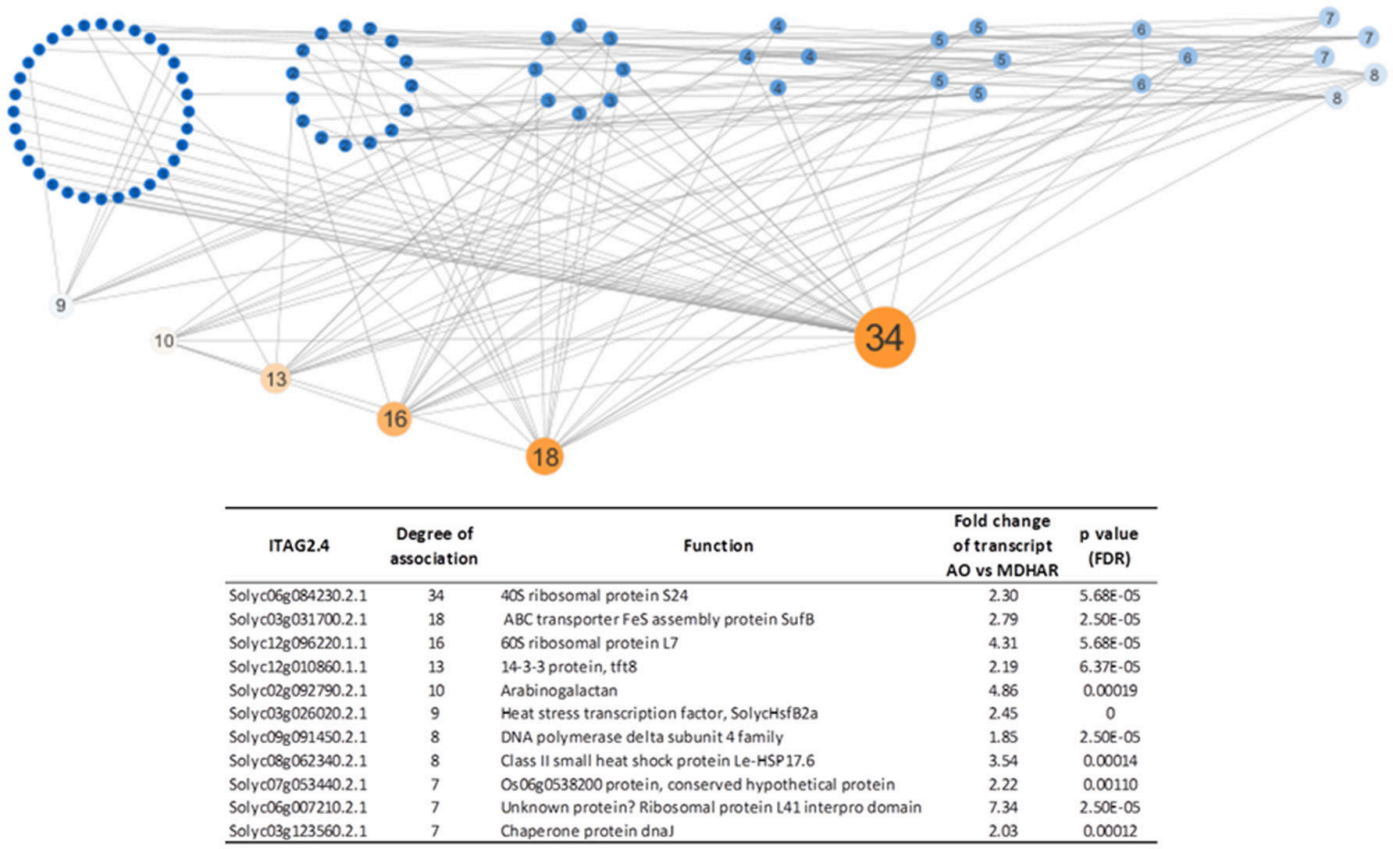
Network analysis (Cytoscape) showing the most highly connected genes based on their expression correlations. A topological overlap matrix was generated using the WGCNA script for the 182 core genes, the 22 metabolites and the 40 proteins. The edge file produced was imported into Cytoscape for network visualization. The network was visualized with a degree-sorted circle layout. The node sizes and node label sizes are proportional to the degree of connectivity of each node as fixed by Cytoscape. The color scale goes from blue (least connected nodes) to orange (most connected nodes). The hub genes are described in the table which gives the most highly connected genes following visualization of the topology overlap matrix, generated by the WGCNA “R” package, with Cytoscape. The ITAG2.4 identifier (Solyc) with the annotated function is shown with the degree of association as defined by Cytoscape. The transcriptome data (fold change between AO and MDHAR RNAi lines with the FDR corrected *p*-value on the transcript levels) for each gene are also shown.

The 182 core gene set was used in network analysis with the metabolites and the protein dataset. A topological overlap matrix using the WGCNA script was produced and an edge file for network visualization using Cytoscape. The network visualized with a degree-sorted circle layout, is shown in Figure 5 with the top eleven hub genes. The most highly connected genes included two or three ribosomal proteins, an observation which is compatible with those above, and two heat-shock proteins (including DnaJ) and a heat-shock transcription factor.

### The transcriptomes show a signature common to stress responses in arabidopsis, particularly mitochondrial stress but no correlation with hydrogen peroxide signaling

**Figure 6:**
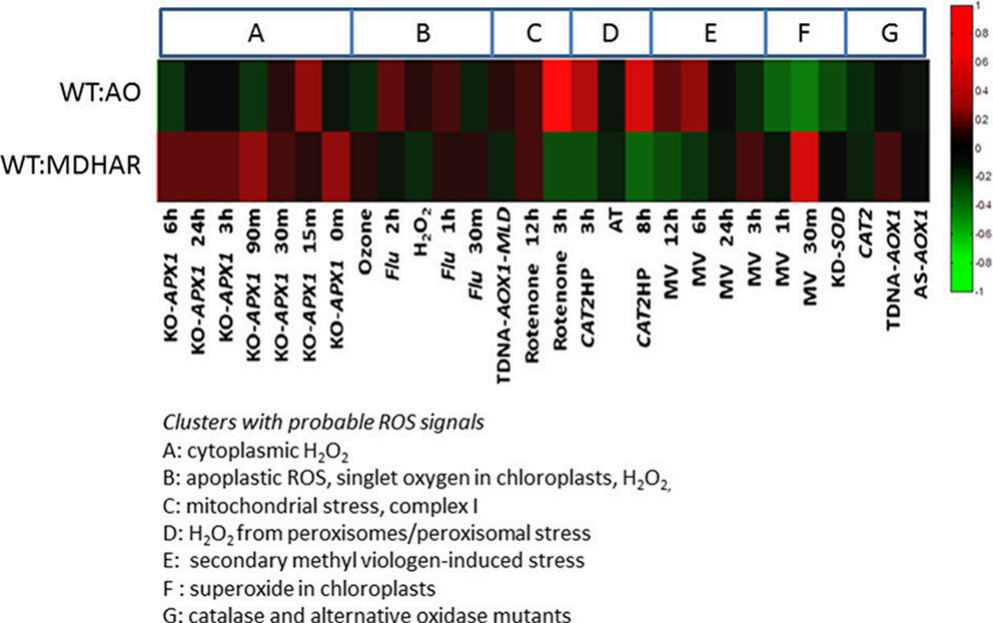
ROSMETER analysis of the WT vs. AO and WT vs. MDHAR transcriptome data from tomato fruit pericarp. Tomato genes were converted to the Arabidopsis homolog and the fold-changes and *p*-values were compared to the Arabidopsis transcriptomes previously described (Rosenwasser et al., 2013) which covered different stress conditions and/or mutant backgrounds in Arabidopsis as clusters as described in the text to the figure. The heatmap generated shows green for general transcriptome down-regulation (opposite fold-change) and red for up-regulation (similar fold-change) in the tomato lines compared to Arabidopsis.

Our tomato transcriptome data was compared using ROSMETER, a bioinformatic tool which unites transcriptome data from different stress experiments or mutants in Arabidopsis linked to specific ROS production in specific organelles (see Materials and methods for more information). Its use is based on the hypothesis that the chemical identity and sub-cellular localization of ROS leave a specific imprint on the transcriptomic response (Rosenwasser et al., 2013). We used the RNAi AO and RNAi MDHAR datasets to compare with the Arabidopsis transcriptomes. Our first observation is that independently of the stress and/or organelle, the transcriptome responses are inversed when these two lines are compared (Figure 6). The particular conditions to be highlighted include mitochondrial stress: in this case, the application of rotenone gives a strong negative correlation with the RNAi MDHAR and a positive correlation with RNAi AO 3 h after application. Negative correlations are also found for RNAi MDHAR with Arabidopsis catalase mutants after 3 and 8 h of high light, whereas the correlations are positive for RNAi AO. Positive correlations between RNAi MDHAR exist with 30 min of methyl viologen and generally the knockout ascorbate peroxidase mutant subjected to high light. Again for RNAi AO, in contrast to RNAi MDHAR, a negative correlation is found for 30 min of methyl viologen treatment.

## Discussion

This study in fruit tissue has revealed some interesting correlations which might provide clues for further research on ascorbate function but some caveats should be pointed out: here we worked on ripening fruit, a non-photosynthetic tissue, where small differences in ascorbate content are observed between the various transgenic lines. The information provided will help decipher the relationships between ascorbate and fruit physiology, especially in fruit subject to biotic and abiotic stresses. However, it remains to be seen if the correlations are more general and an initial question would be to see if they are transferable to other tissues. As a 35S promoter was used it is also possible that the effects we see in fruit are the result of silencing the genes in another part of the plant (for an example of where this is true see our recent results by Truffault et al., 2017). The use of available fruit-specific promoters (Fernandez et al., 2009) and indeed these genes are expressed in different plant organs and fruit tissues (Supplementary Figures S3, S4) would aid in specifically attributing the effects we observed to the fruit. A final caveat is that the correlations we observed are based on microarray data: the 7600 gene tomato microarray is considerably enriched in genes expressed in ripening fruit (Alba et al., 2005; Fei et al., 2011) and therefore well adapted to the purpose of the study. However, global transcriptome analysis using RNA-seq would additionally allow the detection of weakly expressed genes and thus strengthen our conclusions.

### A transcriptional signature is observed and is correlated to silencing of the ascorbate metabolizing enzymes

The transcriptomes revealed a switch linked to the activity of ascorbate oxidase on one side of the switch and the enzymes MDHAR/GLD acting in the other direction, always with wild-type gene expression in the middle. There is a tendency for genes to be up-regulated when ascorbate oxidase is down-regulated. The transcriptome inversion is also seen in datasets from Arabidopsis provided by the ROSMETER tool. The switch seen between AO and MDHAR RNAi lines is capable of distinguishing the conditions found in some of the ROSMETER transcriptome data, although there are limits to this analysis because correlations can be based on a small number of genes (a minimum of 45 according to the original paper). The ROSMETER analysis does not reveal links with hydrogen peroxide metabolism because correlations between our lines and the public data are inversed when comparing ascorbate peroxidase and catalase mutants, both of which in theory should contain more hydrogen peroxide as a result of decreased detoxification. The strong response to rotenone is interesting because rotenone is an inhibitor of mitochondrial complex I, which is also where the galactonolactone dehydrogenase protein is located (Schimmeyer et al., 2016). Also in the paper by Rosenwasser et al. (2013), rotenone-induced stress is also positively correlated with developmental senescence, a process which has parallels with fruit ripening.

The data seem to indicate the existence of a signal affecting transcription closely linked to GLD activity and the redox state of the ascorbate pool. The nature of this signal is unknown but a candidate is monodehydroascorbate, as it is the molecule that links ascorbate oxidase and monodehydroascorbate reductase activity, which could act as a primary signal given (*i*) the short half-life of the molecule, a prerequisite for a role in signaling and (*ii*) the proton transfer that occurs when the radical is reduced back to ascorbate (Bielski et al., 1981) which gives potential for protein modification. Most oxidation reactions with ascorbate are indeed single electron reactions (Heber et al., 1996) generating the radical monodehydroascorbate, this radical is considered to be common and a marker for stress. Another possibility is that redox changes in mitochondria act as a retrograde signal (Rhoads and Subbaiah, 2007); this could be a possibility given the involvement of GLD. Interestingly a dehydroascorbate reductase (DHAR) gene (Solyc05g054760) is one of the 182 differentially regulated genes and is down-regulated in GLD and MDHAR lines and up-regulated in the AO line. Compensatory effects, either by enzymes such as DHAR, or other isoforms of the enzymes under study, cannot be ruled out.

### Ascorbate has links with translation and protein folding

The transcriptional signature and network analysis suggest a role for ascorbate in control of translation and protein folding, at least in the non-photosynthetic tissue studied. Some evidence from the literature has established links between ascorbate and these cellular mechanisms: a good example is the posttranscriptional negative feedback control that ascorbate exerts over its synthesis via the GDP-l-galactose phosphorylase enzyme (Laing et al., 2015) which appears to affect ribosome density on the 5′UTR of the mRNA. Ribosomes and translation are also regulated by stress and redox processes (Halbeisen and Gerber, 2009; Khandal et al., 2009; Mustroph et al., 2009; Gerashchenko et al., 2012; Gismondi et al., 2014; Moore et al., 2016). Protein folding is also affected by environmental challenges: the unfolded protein response results from a period of stress and leads to either up-regulation of proteins required for folding or degradation or in extreme cases cell death if prolonged (Ruberti et al., 2015; Wan and Jiang, 2016). Functional hypotheses such as these would need to be tested. For example, purification of polysomes from the available transgenic lines could lead to information on the RNAs undergoing translation and/or associated proteins and ribosome subunits. Considering the fruit context, this experiment would be especially informative in ripening fruits subjected to high temperature stress or in ripe fruits subjected to stressful postharvest storage conditions that may result in protein mis-folding such as high CO_2_ (Rothan et al., 1997) or chilling temperatures (Stevens et al., 2008).

### Hub gene identification points to novel regulatory roles linked to cellular energy status and signaling

Heat-shock proteins also figure amongst the hub genes identified: three heat-shock proteins are identified: SolycHsfB2a (Solyc03g026020), a Class II small heat-shock protein Le-HSP17.6 (Solyc08g062340) and a chaperone protein DnaJ (Solyc03g123560). Heat-shock proteins are known to be involved in numerous biological phenomena including stress responses, circadian clock regulation, flowering time, and hypocotyl elongation (Charng et al., 2007; Mittler et al., 2012; Kolmos et al., 2014). The hub gene protein DnaJ (hsp40) stimulates the ATPase activity of DnaK (an hsp70 homolog which is interestingly differentially accumulated in the AO and MDHAR proteomes). These proteins bind to unfolded polypeptides to prevent their aggregation, for example under conditions of heat-shock. The DnaK protein is of interest because it has been detected in the proteome of the Arabidopsis mutant for GDP-L-galactose phosphorylase (vtc2), a major control point for ascorbate synthesis, as being up-regulated after 3 and 5 days of high light compared to the wild-type (Giacomelli et al., 2006). This parallels the situation in the AO line (up-regulation) and is opposite to the MDHAR line phenotype (down-regulation) meaning that ascorbate deficiency mirrors the RNAi AO phenotype. Another hub protein, Arabinogalactan, is involved in many biological processes including germination, cell extension, fertilization, binding, and release of calcium and is linked to the auxin response (Ellis et al., 2010). Only one of the hub genes has already been characterized from tomato (Table 7) which is the tft8 protein, a member of the 14-3-3 protein family involved in defense responses (Roberts and Bowles, 1999).

The major phenotypic differences seen in the transgenic lines are related to growth: in previous studies RNAi AO lines tend to show improved yield stability (Garchery et al., 2013) whereas the opposite is true for GLD and MDHAR RNAi lines (Alhagdow et al., 2007; Truffault et al., 2016), a phenomenon which is correlated to the changes in the fruit transcriptomes and could be related to fruit growth. Explanations for the phenotypes might be found in the identities of the hub genes which are broadly involved in signaling translation and protein (re)folding. We also noted the presence of many ATP-dependent proteins. The yield phenotypes could therefore reflect differences in cellular energy levels.

### Differences at the protein and metabolite level do not follow transcriptional differences and show a second level of control

As many studies have observed, RNA and protein abundance are often weakly correlated (Halbeisen and Gerber, 2009; Petricka et al., 2012) and our data corroborate this: the quantity of the differentially accumulated proteins is generally not linked to their transcriptional levels (Table 6). For the observable proteins, the GLD proteome is not significantly affected: the GLD proteome actually is remarkably visually similar to the AO proteome which is surprising as the transcriptome data showed contrasted differential expression of genes for these two lines. The metabolic profile is similar to the proteome profile in that several metabolites show opposite changes compared to wild-type between AO and MDHAR RNAi lines, this includes dehydroascorbate, chlorogenic acid, and alanine. The changes seen in dehydroascorbate are counter-intuitive when compared to what is expected based on enzyme activity. We, with other independent groups, have previously reported increases in ascorbate levels on reduction of MDHAR activity (a peroxisomal and cytosolic isoform; Haroldsen et al., 2011; Gest et al., 2013a) which implies feedback regulation. Links have been shown between polyphenols such as chlorogenic acid or anthocyanins and ascorbate (Page et al., 2012); also oxidation of chlorogenic acid is correlated with oxidation of ascorbate to dehydroascorbate (Takahama, 1998) and ascorbate can regenerate chlorogenic acid from its corresponding radicals (Yamasaki and Grace, 1998). Our results show less chlorogenic acid in lines with lower ascorbate oxidase. We should point out that the genotype used in this study (West Virginia 106) is particularly rich in chlorogenic acid, the wild-type contains 3.25 mg/g dwt of chlorogenic acid whereas reported concentrations range from 0.24 to 3.69 mg/100 g fresh weight (between ~0.024 and 0.37 mg/g dwt; Slimestad and Verheul, 2005; Peng et al., 2008). The differences in alanine are also of interest as this metabolite is produced directly from pyruvate and therefore has close links with glycolysis, the oxidative pentose phosphate pathway and the TCA cycle and cellular energy production. Alanine is a product of anaerobic metabolism resulting from high rates of amino acid interconversion (Rocha et al., 2010b). Unlike lactate and ethanol, alanine accumulation does not have detrimental side effects but maintains the glycolytic flux (Rocha et al., 2010a).

In conclusion, this study in a non-photosynthetic tissue reveals that the activity of enzymes controlling the oxidation state of the ascorbate pool are correlated to a few metabolic changes and some changes in protein quantity, particularly those involved in protein folding. The relatively low number of metabolic changes compared to gene expression probably reflects buffering of the system. In addition to this post-transcriptional regulation, a transcriptional response is seen which is also linked to the activity of the final biosynthesis enzyme, galactonolactone dehydrogenase. Plants silenced for this enzyme show a transcriptional response similar to that of MDHAR silenced plants and opposite to ascorbate oxidase silenced plants implying signaling from the ascorbate pool. Network analysis reveals hub genes involved in fundamental cellular mechanisms including cellular signaling, translation and protein synthesis as well as the heat-shock response.

## Author contributions

RS: supervised the experiments, carried out the bioinformatic analysis of the data and wrote the article with the contribution of all the authors; MF and J-PB: carried out the proteome study; PB, CD, MM, AM, and J-LP: carried out the metabolic analyses; VG and JP: carried out the microarrays and transcriptome data normalization; ClD: analyzed the ROSMETER results; CG: carried out the greenhouse measurements, sampling and provided technical assistance to RS; BG and DP: carried out the post-harvest study; MC, HG, and VT: helped with the writing; CR: conceived the project and obtained the funding. All authors approved the final version of the manuscript.

### Conflict of interest statement

The authors declare that the research was conducted in the absence of any commercial or financial relationships that could be construed as a potential conflict of interest.

## Supplementary material

Supplementary Figure S1 Heatmap showing protein levels in pericarp of fruit 20 days after anthesis (3 pools of 30 fruits) of the transgenic lines and wild-type. All proteins were separated by two-dimensional gel electrophoresis and identified by mass spectrometry. The log2 of the protein ratio with the wild-type for each transgenic line is presented. The scale goes from green (protein decreased compared to wild-type) to purple (protein increased). A comparison of the means was carried out using a Kruskal Wallis test with correction (Dunn). Different letters indicate significant differences (5% significance level). SGN codes are found at https://solgenomics.net/.

Supplementary Figure S2 WGCNA module identification. A graphical representation of the data from Supplementary Table S4: module eigengenes are labeled by color and the module membership scale from red (high adjacency, positive correlation) to green (low adjacency, negative correlation) shown for each metabolite and protein. The 7633 genes were used to generate the topology overlap matrix. SGN codes are found at https://solgenomics.net/.

Supplementary Figure S3 Gene expression profiles for AO, Solyc04g054690; GLD, Solyc10g079470 and MDHAR, Solyc09g009390 as obtained from the TomExpress tool (http://tomexpress.toulouse.inra.fr).

Supplementary Figure S4 Gene expression profiles for AO, Solyc04g054690; GLD, Solyc10g079470 and MDHAR, Solyc09g009390 as obtained from the Solgenomics expression atlas tool (http://tea.solgenomics.net).

Supplementary Table S1 Ascorbate and dehydroascorbate levels in pericarp fruit 20 days after anthesis of transgenic lines and WT. Ascorbate and dehydroascorbate were assayed in the pericarp tissue (3 biological replicates of 30 fruits per pool) of the lines by a spectrophotometric method. Measurements show means with standard error (SE). A comparison of means was carried out using a Kruskal Wallis test with correction (Dunn). Different letters indicate significant differences (5% significance level).

Supplementary Table S2 Fruit metabolite data (orange fruit pericarp). Quantitative data for the following metabolites: galactose, mannose, glutamate, glutamine, alanine, aspartate, tyrosine, fructose, glucose, sucrose, ascorbate, dehydroascorbate, GABA, citramalate, citrate, malate, chlorogenic acid, caffeic acid glucoside, cis-chlorogenic acid, quercetin derivative, and rutin as measured by specific methods (^1^H-NMR for polar compounds, HPLC-DAD for the major polyphenols, spectrophotometrically for ascorbate and dehydroascorbate) for each of the three pools for the wild type, AO, GLD and MDHAR RNAi lines.

Supplementary Table S3 Proteins. Raw data for quantifiable proteins (see Materials and methods) from orange pericarp with spot identifier code for each of the three pools for the wild type, AO, GLD, and MDHAR RNAi lines.

Supplementary Table S4 WGCNA. Solyc identifier, gene function, GO terms, module color, module membership (MM or eigengene-based connectivity) and associated *p*-value for the 38 modules and 7633 genes as generated by the WGCNA R package.

Supplementary Table S5 Transcriptome raw data. For each of the 7633 genes mean expression (normalized) in each of the three transgenic lines and wild type with standard error (SE). F values, *p*-values, false discovery rate corrected *p*-values and fold change (FC) are also given for each comparison as follows: 1: AO vs. WT, 2: GLD vs. WT, 3: MDHAR vs. WT, 4: AO vs. MDHAR, 5: AO + MDHAR/(2 × WT) = myModel, 6: AO vs. GLD, 7: GLD vs. MDHAR, 8: AO + GLD/(2 × WT) = myModel2, 9: GLD + MDHAR/(2 × WT) = myModel3.

## Abbreviations

RNAi: RNA interference
GLD: galactonolactone dehydrogenase
AO: ascorbate oxidase
MDHAR: monodehydroascorbate reductase
GO: Gene Ontology
FDR: false discovery rate
WGCNA: Weighted gene correlation network analysis
WT: wild-type
TCA: tricarboxylic acid cycle
HSP70: heat-shock protein family
ATP: adenosine triphosphate.
