# Supplementary figures and images for: A Systems Biology Study in Tomato Fruit Reveals Correlations between the Ascorbate Pool and Genes Involved in Ribosome Biogenesis, Translation, and the Heat-Shock Response
Source: Front Plant Sci. 2018 Feb 14;9:137. doi: 10.3389/fpls.2018.00137 (PMC5817626; doi:10.3389/fpls.2018.00137)

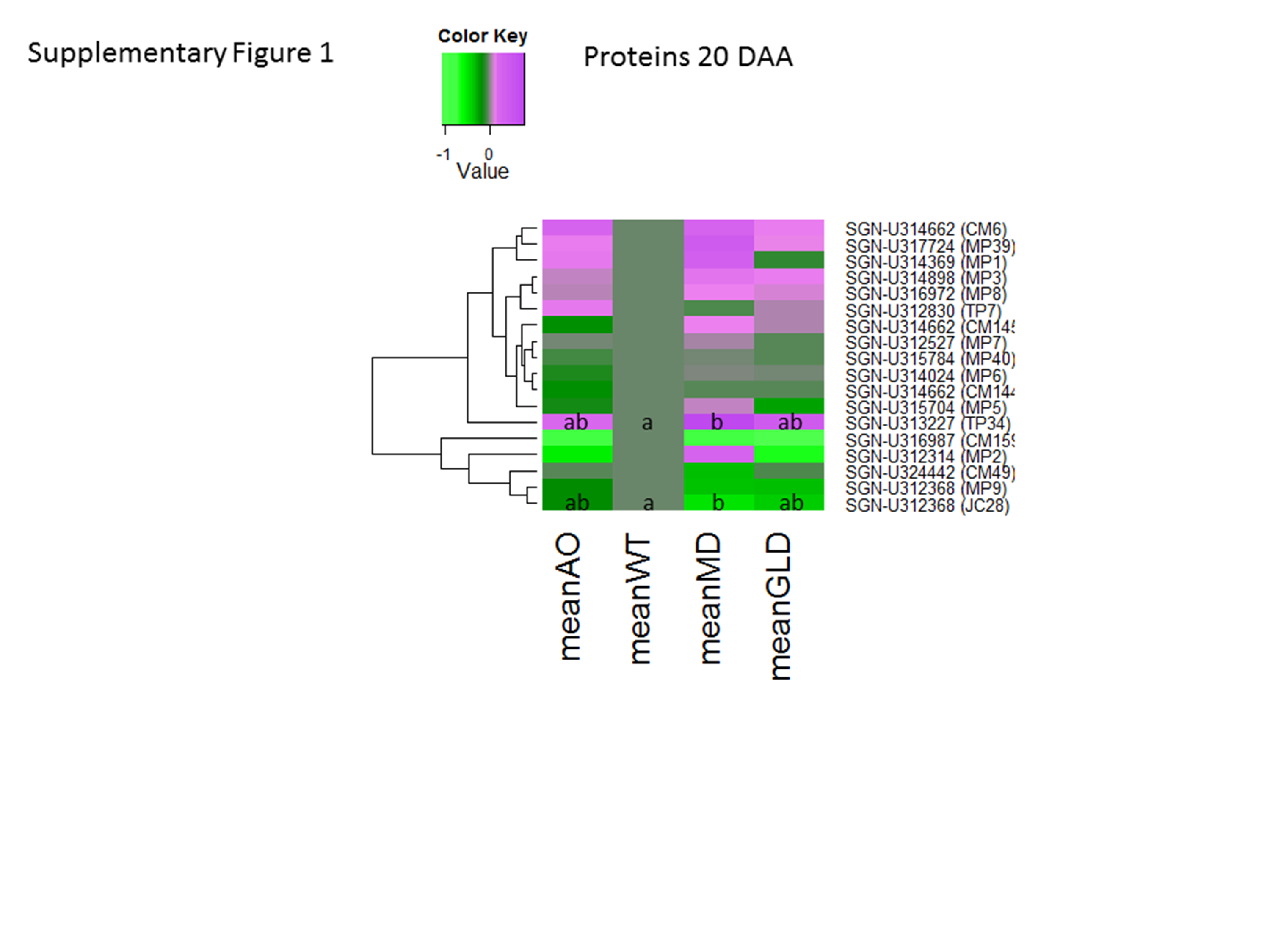

Supplement: Supplementary Figure S1 — Heatmap showing protein levels in pericarp of fruit 20 days after anthesis (3 pools of 30 fruits) of the transgenic lines and wild-type. All proteins were separated by two-dimensional gel electrophoresis and identified by mass spectrometry. The log2 of the protein ratio with the wild-type for each transgenic line is presented. The scale goes from green (protein decreased compared to wild-type) to purple (protein increased). A comparison of the means was carried out using a Kruskal Wallis test with correction (Dunn). Different letters indicate significant differences (5% significance level). SGN codes are found at https://solgenomics.net/. [file Image1.TIF]

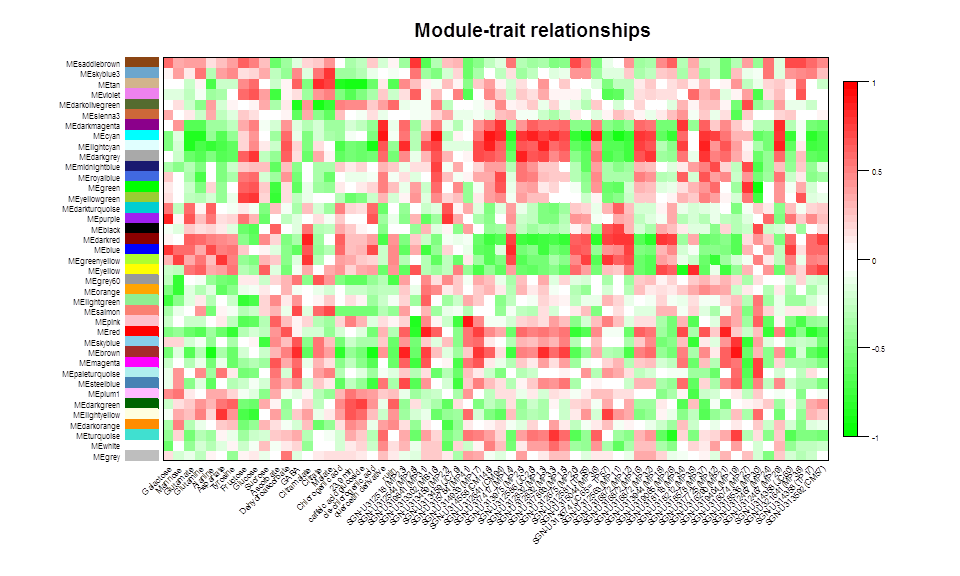

Supplement: Supplementary Figure S2 — WGCNA module identification. A graphical representation of the data from Supplementary Table S4: module eigengenes are labeled by color and the module membership scale from red (high adjacency, positive correlation) to green (low adjacency, negative correlation) shown for each metabolite and protein. The 7633 genes were used to generate the topology overlap matrix. SGN codes are found at https://solgenomics.net/. [file Image2.PNG]

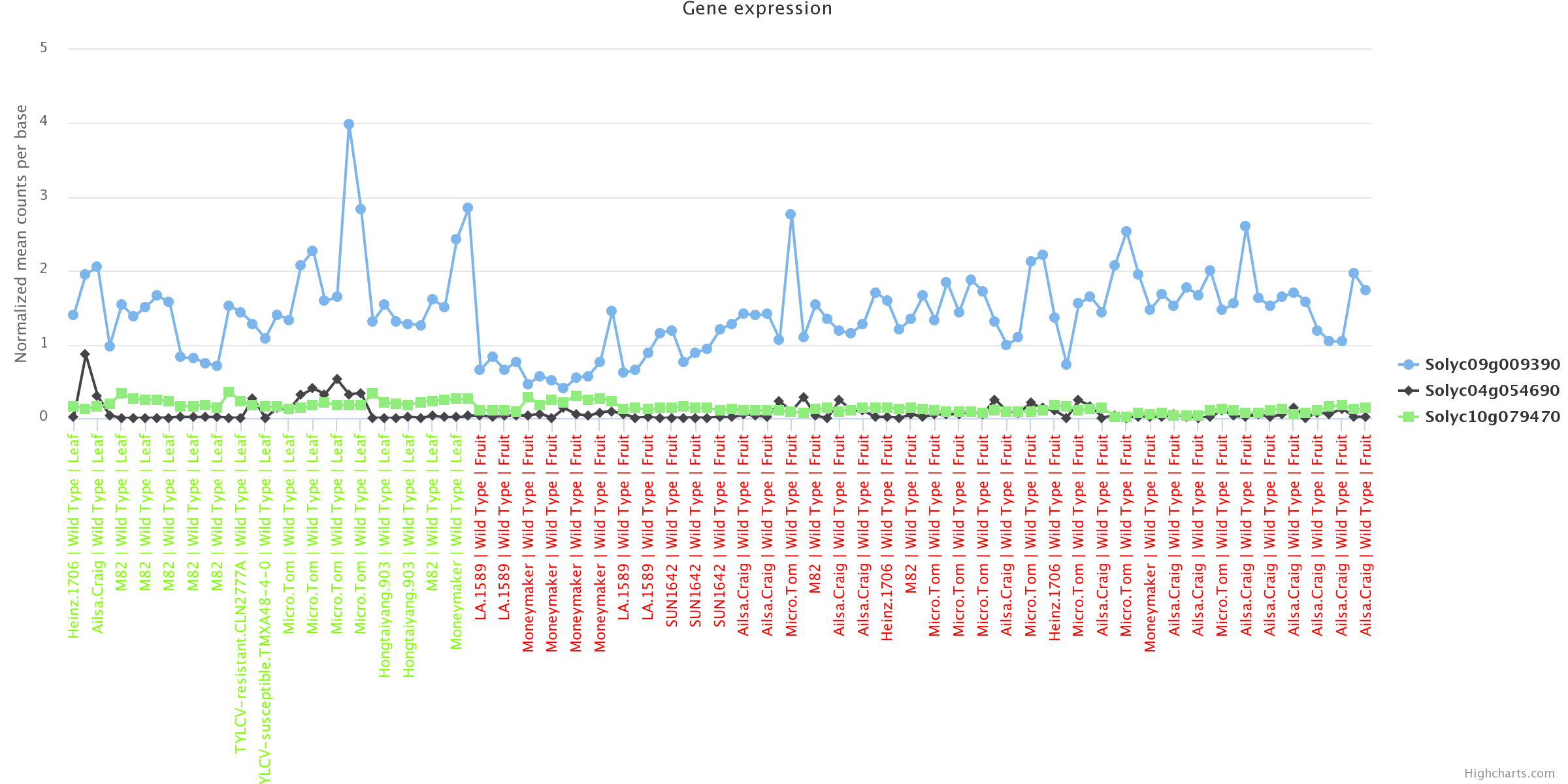

Supplement: Supplementary Figure S3 — Gene expression profiles for AO, Solyc04g054690; GLD, Solyc10g079470 and MDHAR, Solyc09g009390 as obtained from the TomExpress tool (http://tomexpress.toulouse.inra.fr). [file Image3.PNG]

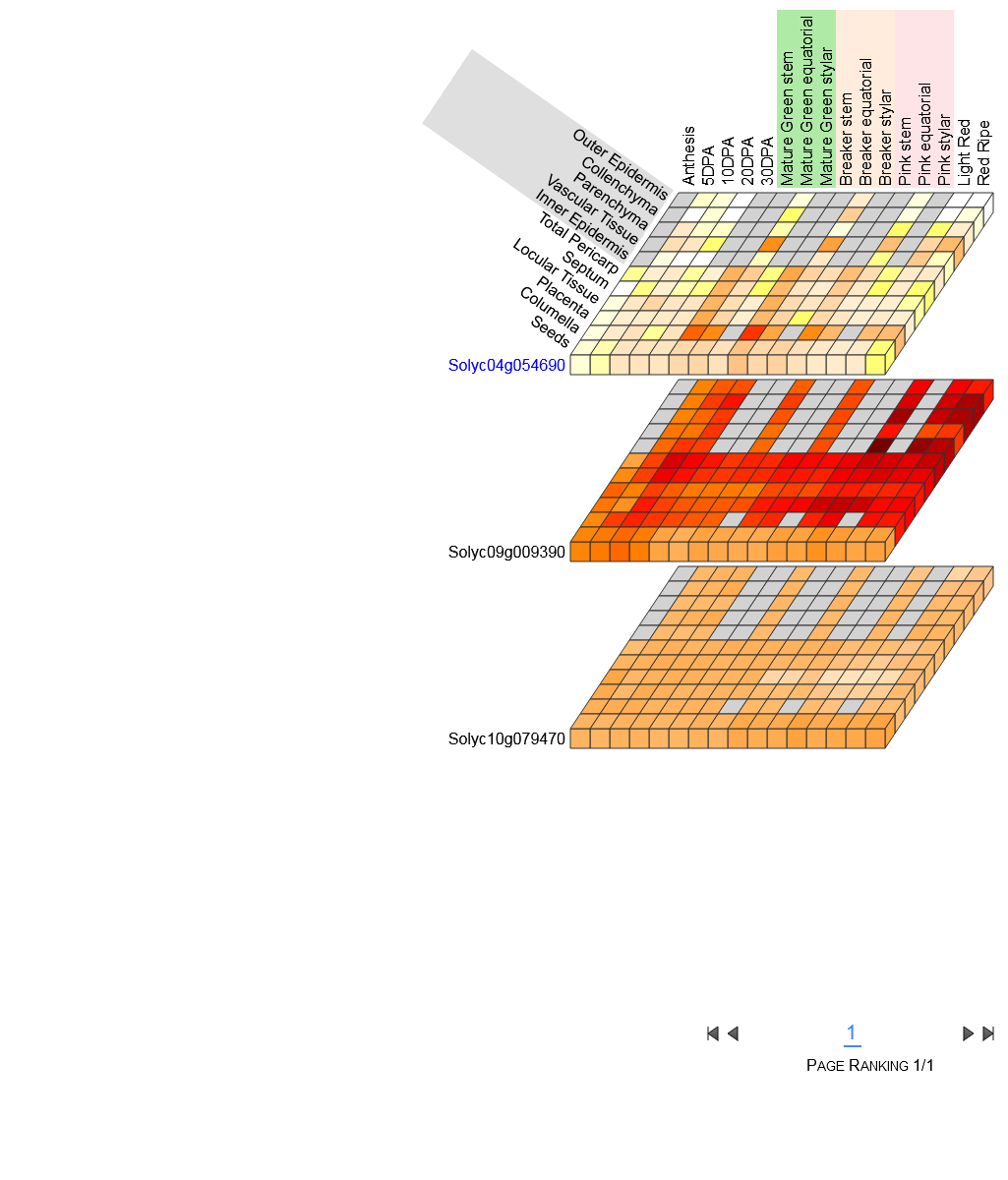

Supplement: Supplementary Figure S4 — Gene expression profiles for AO, Solyc04g054690; GLD, Solyc10g079470 and MDHAR, Solyc09g009390 as obtained from the Solgenomics expression atlas tool (http://tea.solgenomics.net). [file Image4.PNG]
