# Supplementary material for: A Systems Biology Study in Tomato Fruit Reveals Correlations between the Ascorbate Pool and Genes Involved in Ribosome Biogenesis, Translation, and the Heat-Shock Response
Source: Front Plant Sci. 2018 Feb 14;9:137. doi: 10.3389/fpls.2018.00137 (PMC5817626; doi:10.3389/fpls.2018.00137)
Supplement: Supplementary Table S1 — Ascorbate and dehydroascorbate levels in pericarp fruit 20 days after anthesis of transgenic lines and WT. Ascorbate and dehydroascorbate were assayed in the pericarp tissue (3 biological replicates of 30 fruits per pool) of the lines by a spectrophotometric method. Measurements show means with standard error (SE). A comparison of means was carried out using a Kruskal Wallis test with correction (Dunn). Different letters indicate significant differences (5% significance level). [file DataSheet1.pdf]

Supplementary data.

Supplementary Figure S1: Heatmap showing protein levels in pericarp of fruit 20 days after anthesis (3 pools of 30 fruits) of the transgenic lines and wild-type.

All proteins were separated by two-dimensional gel electrophoresis and identified by mass spectrometry. The log2 of the protein ratio with the wild-type for each transgenic line is presented. The scale goes from green (protein decreased compared to wild-type) to purple (protein increased). A comparison of the means was carried out using a Kruskal Wallis test with correction (Dunn). Different letters indicate significant differences (5% significance level). SGN codes are found at https://solgenomics.net/.

Supplementary Figure S2: WGCNA module identification

A graphical representation of the data from Supplementary Table SIV: module eigengenes are labeled by colour and the module membership scale from red (high adjacency, positive correlation) to green (low adjacency, negative correlation) shown for each metabolite and protein. The 7633 genes were used to generate the topology overlap matrix. SGN codes are found at https://solgenomics.net/.

Supplementary Table SI: Ascorbate and dehydroascorbate levels in pericarp fruit 20 days after anthesis of transgenic lines and WT.

Ascorbate and dehydroascorbate were assayed in the pericarp tissue (3 biological replicates of 30 fruits per pool) of the lines by a spectrophotometric method. Measurements show means with standard error (SE). A comparison of means was carried out using a Kruskal Wallis test with correction (Dunn). Different letters indicate significant differences (5% significance level).

Ascorbate in green fruit

	**reduced AsA (mg/100gfwt)**	SE	p	**DHA (mg/100gfwt)**	SE	p
WT	11.31	1.02	a	7.18	0.89	a
AO	9.80	1.08	a	8.45	0.34	a
GLD	10.72	0.50	a	6.92	0.38	a
MD	13.85	0.28	a	7.15	0.24	a

Supplementary Table SII: Fruit metabolite data (orange fruit pericarp). *File « agrobiM_orange_withGLD_andpp.csv »*

Quantitative data for the following metabolites: galactose, mannose, glutamate, glutamine, alanine, aspartate, tyrosine, fructose, glucose, sucrose, ascorbate, dehydroascorbate, GABA, citramalate, citrate, malate, chlorogenic acid, caffeic acid glucoside, cis-chlorogenic acid, quercetin derivative and rutin as measured by specific methods (^{1}H-NMR for polar compounds, HPLC-DAD for the major polyphenols, spectrophotometrically for ascorbate and dehydroascorbate) for each of the three pools for the wild type, AO, GLD and MDHAR RNAi lines.

Supplementary Table SIII: Proteins. *file « agrobiP_orange_withGLD.csv »*

Raw data for quantifiable proteins (see Materials and methods) from orange pericarp with spot identifier code for each of the three pools for the wild type, AO, GLD and MDHAR RNAi lines.

Supplementary Table SIV: WGCNA. *file « geneInfo_withGLD.xls »*

Solyc identifier, gene function, GO terms, module colour, module membership (MM or eigengene-based connectivity) and associated p-value for the 38 modules and 7633 genes as generated by the WGCNA R package.

Supplementary Table SV: Transcriptome raw data. *file "agrobi_maanova_analysis_withGLD_sep15"*

For each of the 7633 genes mean expression (normalized) in each of the three transgenic lines and wild type with standard error (SE). F values, p-values, false discovery rate corrected p-values and fold change (FC) are also given for each comparison as follows: 1: AO versus WT, 2: GLD versus WT, 3: MDHAR versus WT, 4: AO versus MDHAR, 5: AO + MDHAR/(2xWT) = myModel, 6: AO versus GLD, 7: GLD versus MDHAR, 8: AO + GLD/(2xWT) = myModel2, 9: GLD + MDHAR/(2xWT) = myModel3.
